# Mental health implications in the older adults of internet access in China

**DOI:** 10.3389/fpubh.2026.1858590

**Published:** 2026-08-25

**Authors:** Chi Zhang, Lue Zhou, Xiaoyu Cheng

**Affiliations:** Zhejiang University of Finance and Economics, Hangzhou, China

**Keywords:** active aging, China, older adults’ mental health, internet access, social networks

## Abstract

Social isolation is a significant factor contributing to mental health issues among the older adults, particularly in societies experiencing rapid population aging. Notably, digital technology offers an effective means to address this issue. However, little is known that how access to internet affects the mental health of the older adults. Based on the China Longitudinal Aging Social Survey (2018–2020) data, a national representative data, this study explored the impact of internet access on the older adults mental health by broadening their social network. The results show that internet access significantly reduces the risk of depression of the older adults. This mitigating effect is attributed to the way digital technology enhances the ties of the older adults with family and friends, which in turn helps prevent social isolation and leads to improved mental health. Furthermore, the study finds that community-based service infrastructure and family-level filial support can significantly amplify the effect of internet access on mental health. The effects are stronger for urban residents and “young-old” cohort (aged 60–80). These findings demonstrate that fostering digital inclusion among the older adults is an effective strategy for enhancing their psychological wellbeing within aging societies.

## 1 Introduction

With accelerating global population aging, mental health illness of the older adults has become increasingly prominent, emerging as a critical challenge in aging societies worldwide (1). Global projections indicate that the proportion of people aged 65 and above will rise from 10% in 2022 to 16% by 2050 (2). This demographic shift has precipitated formidable mental health challenges: approximately 15% of the global older adults population is experiencing varying degrees of psychiatric disorders, with mental illness-related disability accounting for over 6% of total disability burden within this age cohort (3). In China, the situation is particularly acute, with surveys indicating that 26.4% of the older adults exhibit depressive symptoms of varying severity (4). As one of the world’s most populous nation, China is experiencing particularly rapid demographic aging. The proportion of the population aged 65 and above in China increased from 7% in 2000, marking the country’s entry into an aging society, to a projected 15.6% by the end of 2024.^1^ This represents a more than twofold increase over the 24-year period, with the absolute number of this demographic expected to exceed 220 million (5). Physiological decline associated with aging increases the older adults’s exposure to stressors that threaten their mental health, thereby undermining their overall psychological wellbeing. Statistics show that nearly half of the older adults aged 65 to 84 have experienced mental health issues, including depression, loneliness, anxiety, and stress (6). Depression not only severely impairs the quality of life for the older adults but is also associated with higher mortality and morbidity rates (7). From a health economics perspective, the older adults face a high depreciation rate of health capital and inherently fragile psychological resilience (8). This fragility is often exacerbated by a reduction in social support due to shrinking social networks in later life, highlighting the pressing need for alternative channels to sustain social connectedness.

**Figure 1:**
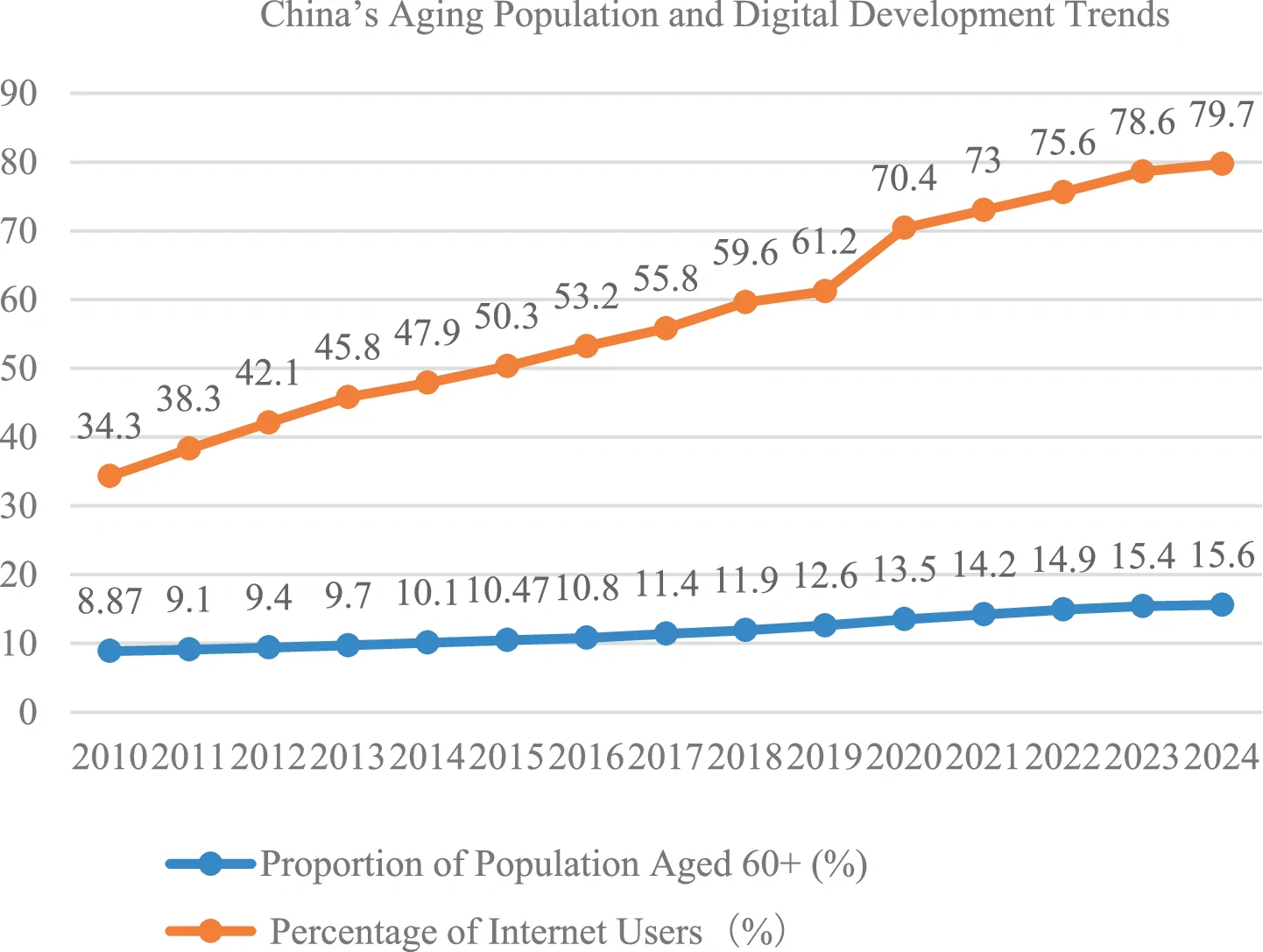
Rising trends in China’s aging population and Internet users (2010–2024). (1) The blue line (left axis) represents the proportion of the population aged 65 and above, while the orange line (right axis) indicates the percentage of Internet users. (2) Data for 2019 is based on the June 2019 survey results (the 44th CNNIC report) due to the unavailability of year-end data; all other years represent year-end statistics. (3) Data sources: China Internet Network Information Center (CNNIC) and the National Bureau of Statistics of China (NBS). (4) For visualization clarity, a dual-axis scale is employed.

**Figure 2:**
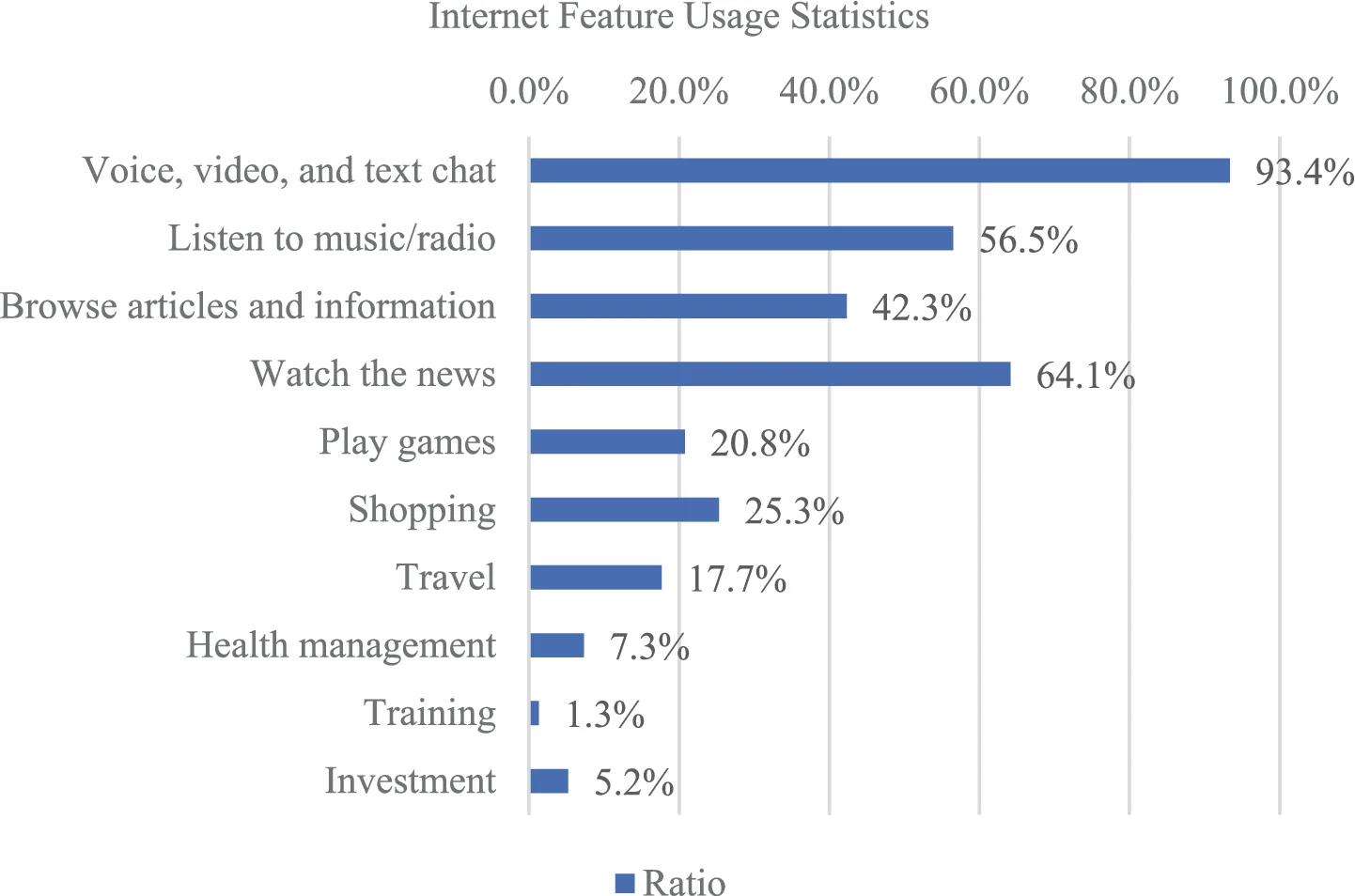
Internet function usage among Chinese older adults population in 2020. The data illustrates the proportion of older adults users engaging in various online activities, reflecting a high concentration in social communication. Data source: The China Longitudinal Aging Social Survey (CLASS) 2020.

Meanwhile, the parallel processes of aging and digital transformation are creating new opportunities for mental health interventions among older adults. As shown in Figure 1, which summarizes these concurrent trends, China, as one of the world’s fastest-developing digital economies, has seen its internet penetration rate continue to rise. As of June 2025, internet users aged 60 and above reached 161 million, with the percentage of Internet users within this age group reaching 52% (9). The rapid advancement of digital technology facilitates the older adults’s access to information and public services. The China Longitudinal Aging Social Survey (CLASS) show that social interaction is a primary motivation for internet access among the Chinese older adults. As shown in Figure 2, voice, video, and text chat (93.4%) is the dominant online activity, underscoring that their engagement with digital technology is fundamentally social. By maintaining emotional connections with family and friends through online platforms, the older adults can secure more robust social support, which theoretically alleviates loneliness and promotes psychological wellbeing (10).

Previous studies have found that internet access promotes mental health by reducing depression and loneliness (11, 12). The internet access can enhance their social participation (13–15), strengthened social support (16–18), improved health behaviors (19–21), and increased information accessibility (22, 23), thereby improving mental health of the older adults. While some studies have found that excessive or inappropriate use of internet can decrease social trust and compromise psychological wellbeing (24). Several recent studies using nationally representative data from China have made important contributions to this literature. Lu (25) employs CHARLS 2020 cross-sectional data to examine how increasingly specific dimensions of internet use affect depressive symptoms. Using multiple linear regression and propensity score matching, Lu (25) finds that deeper internet engagement reduces depression, with significant heterogeneity across disability, education, and residence. Wang and Shi (26) establish causality using panel data and find that internet use benefits older adults lacking intergenerational support or suffering from chronic diseases. Qiu et al. (27) adopt a mixed-methods approach using CHARLS 2018 data to examine how internet usage frequency, device types, and online activities affect body pain and loneliness, complemented by qualitative interviews with six older adults during the COVID-19 pandemic. Despite their valuable contributions, none of these studies have systematically grounded their analyses in social network theory to unpack the relational mechanisms through which internet access affects mental health. Moreover, existing studies have implicitly assumed that the benefits of internet access are relatively uniform once access is granted, focusing on identifying who benefits more (e.g., disabled, less educated, or chronically ill older adults) without systematically examining what contextual factors enable or inhibit the realization of these benefits. Besides, while existing studies have addressed endogeneity to varying degrees, their treatments remain partial or absent.

This study aims to systematically investigate the impact of internet access on improving mental health among the older adults through the social network pathway. Given that China is experiencing rapid population aging alongside digitalization, investigating this issue in the Chinese context is of significant practical relevance. This study addresses three primary research questions: Does internet access effectively improve mental health among the older adults? What role do social networks play in the mental health effects of internet access? To what extent do family care and community resources for the older adults moderate the effectiveness of internet access in improving mental health? The empirical analysis employs a fixed-effects model using data from the 2018–2020 China Longitudinal Aging Social Survey (CLASS), a nationally representative dataset.

The research design is structured around four key components. First, we measure key variables: internet use (captured through both a binary indicator and nuanced social function usage) and mental health (assessed via the CES-D scale, where higher scores indicate more severe depressive symptoms). Second, to establish causal inference, we employ a set of rigorous econometric strategies to address potential endogeneity and ensure robustness. Third, we empirically examine the underlying mechanisms, focusing on pathways through personal (family/friend) and community-level social networks, while also testing the moderating roles of formal and informal older adults care support. Finally, we conduct heterogeneity analyses across residential (urban/rural) and age (young–old/old–old) subgroups to explore differential effects.

This research contributes to the literature in three ways. Firstly, this study, from the perspective of social network theory, analyzes the internal mechanism by which internet access affects the mental health of the older adults. In particular, by distinguishing between friend networks, which are voluntary and sustained through active communication, and family networks, which are obligatory and constrained by kinship structure, this study identifies the differentiated pathways through which the internet strengthens distinct network types and their varying implications for mental health. This refines the technological protection hypothesis by specifying the relational channels through which digital engagement benefits psychological wellbeing. Second, this study systematically examines how the mental health returns to internet access are constrained by family care and community support resources. This finding extends the digital divide framework from a narrow concern with access to a broader focus on effectiveness in use, demonstrating that the benefits of internet access are not uniform but depend on the availability of offline caregiving and community contexts in which older adults are embedded. Third, this study strengthens causal inference by jointly addressing self-selection, simultaneity, and omitted variable bias through fixed effect model, propensity score matching, and instrumental variable estimation. This multi-method approach provides more credible causal warrant than the single-strategy designs typically employed in the literature.

## 2 Literature review and conceptual framework

Social networks are defined as dynamic structures of relationships and interactions between individuals and society within specific cultural contexts (28). As the bonds through which individuals are embedded in social structures, they serve as vital sources of emotional support and social capital (29). Extensive research has confirmed that social networks play a pivotal role in the health and wellbeing of the older adults (30, 31). Within this domain, it is important to distinguish social networks from related constructs such as social support and social participation: social networks refer to the structural properties of relational ties, whereas social support and social participation reflect the functional and behavioral dimensions embedded within these structures (32). This study focuses on network structure per se, because internet access, by altering the composition and reach of the older adults’s relational ties, may reconfigure the channels through which support and participation are subsequently mobilized. However, with evolving social environments and changing family structures, this positive influence faces the risk of erosion in contemporary societies. Particularly in the context of Chinese modernization, large-scale population mobility and the transformation of family structures have disrupted traditional intergenerational living arrangements, typically characterized by multi-generational co-residence, significantly weakening family support networks and exposing an increasing number of Chinese older adults to the risk of social isolation (33).

Meanwhile, the internet has fundamentally reshaped social interaction across all demographics, serving as a powerful platform for maintaining relationships beyond physical proximity. For the older adults, this technology has gradually emerged as a vital tool to overcome social isolation. By transcending temporal and spatial constraints, it provides new communication channels and avenues for social participation, compensating for deficits in traditional social connections while also reshaping the older adults’s social interaction patterns to some extent (34). This technological intervention enables the older adults to reestablish connections and identity within multi-layered social networks, thereby creating new possibilities for improving their mental health (63). However, different types of social relationships vary in structure and function, suggesting that the internet may play distinct roles across different social networks (35).

From the perspective of social network multidimensionality, the internet’s embeddedness patterns and effects differ between friend networks and family networks. The convoy model of social relations conceptualizes social networks as dynamic, multilayered structures surrounding individuals across the life course, with family members typically occupying the innermost circle and friends and community ties distributed across outer layers (36). This theory differentiates family networks from friendship networks, providing a basis for understanding how internet access strengthens these social networks at different levels. Family networks, primarily composed of children, spouses, and other relatives, are often constrained by structural factors such as the number of children and represent stable social networks (36). Within this structure, internet integration broadens communication pathways between the older adults and family members (37). Through instant messaging and video calls, the older adults can engage in interactive communication with relatives across temporal and spatial barriers, effectively alleviating emotional distance caused by physical separation and thereby strengthening their social networks (38). In contrast, friend networks usually include friends, neighbors, or colleagues, and they differ from family ties in important ways. These connections are often less formal and require regular interaction to be sustained, which makes them relatively fragile (39). This fundamental difference between reinforcing stable kin ties and activating voluntary peer ties explains why internet access may affect the size and quality of family and friend networks in divergent ways.

Beyond immediate interpersonal ties, the internet plays a crucial role in promoting community-level social participation. As the older adults experience physical decline and reduced mobility, their daily activity space narrows, and the community gradually becomes the central place of life. In this setting, the internet serves not only as a source of information about local affairs but also as a practical means of participation. For instance, through online platforms, many older adults individuals encounter notices of community programs and sometimes join activities that take place either online or face-to-face. Such experiences can help them expand their social networks and, to some degree, strengthen their sense of belonging (40). In this way, community-based participation may ease psychological loneliness by offering support that the family alone cannot provide.

**Figure 3:**
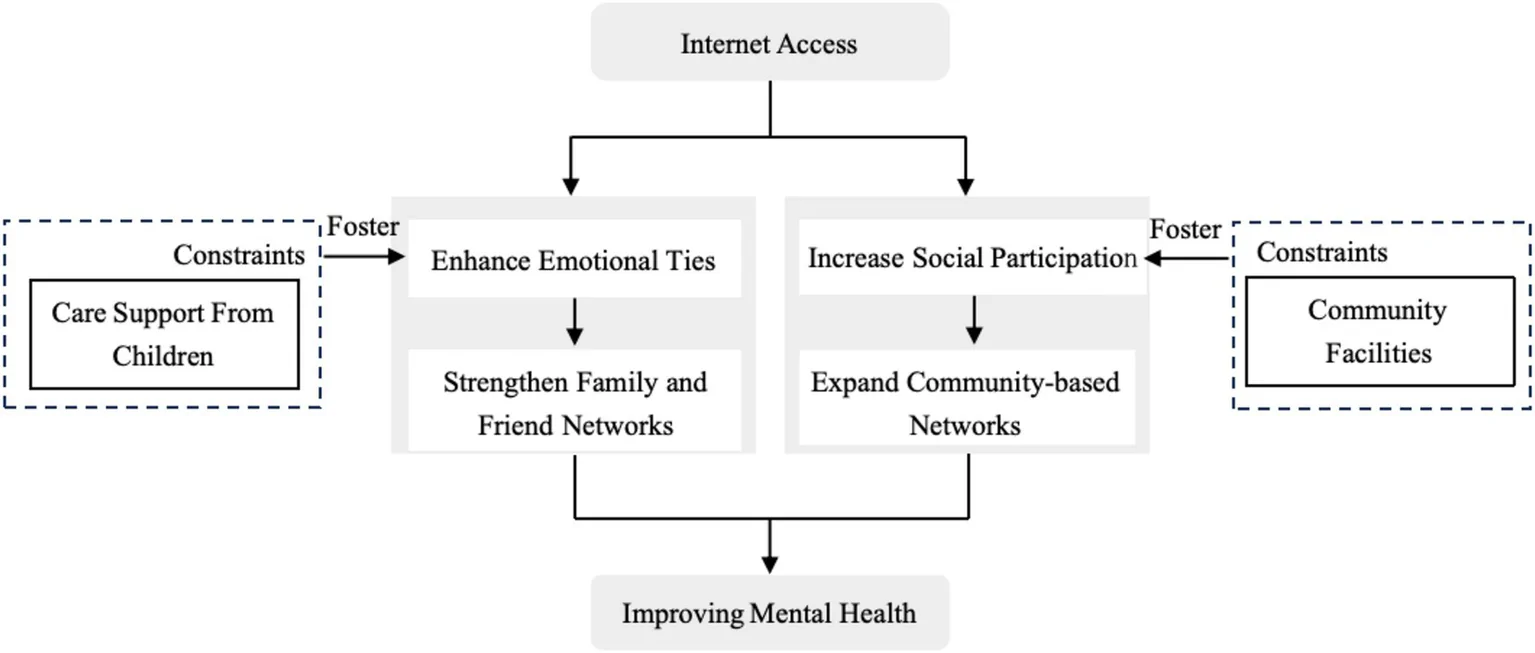
Research framework. Source: Authors own work.

The enabling effects of internet access within family and community networks are significantly contingent upon specific contextual conditions. At the family level, the marginal utility of internet communication is lower when children provide sufficient in-person care. Conversely, in the absence of proximate family support, the internet becomes a critical supplementary conduit for maintaining emotional connections. At the community level, online engagement more readily facilitates active participation in communities with ample resources and organized activities; in resource-poor settings, however, similar opportunities frequently do not lead to substantive involvement (Figure 3).

Collectively, the internet contributes to the older adults’s mental health by transforming their social networks along two interconnected pathways: reinforcing personal networks, especially intergenerational family ties, and facilitating the development of community networks through enhanced social participation. The efficacy of these network-based mechanisms is crucially moderated by the family and community environments in which the older adults are situated. Accordingly, we propose the following hypotheses:

H1: Internet access improves the mental health of the older adults by strengthening and expanding their social networks.

H1a: Internet access produces differentiated effects across social network dimensions, with a stronger effect on friendship networks than on family networks.

H2: The positive effect of internet access on mental health can be moderated by the older adults’s family informal care and community-based service infrastructure, but these moderating effects are heterogeneous.

## 3 Data and methods

### 3.1 Data

This study utilizes data from the China Longitudinal Aging Social Survey (CLASS), a nationally representative longitudinal survey of Chinese older adults aged 60 and older. CLASS employs a multistage stratified probability-proportional-to-size (PPS) sampling design: county-level administrative districts served as primary sampling units (PSUs) and villages/residents’ committees as secondary sampling units (SSUs). Within each SSU, household addresses formed the sampling frame from which eligible households were randomly selected, and one individual aged 60 or older per household was randomly chosen for a face-to-face interview. The 2014 baseline survey, conducted from May to November 2014, completed 11,511 individual questionnaires across 462 SSUs in 30 provinces, with quality control ensured through on-site supervision, remote data analysis, and telephone verification. The 2018 and 2020 follow-up waves adopted the same sampling framework.

This study primarily uses data from the 2018 and 2020 waves. These two waves were chosen because, although the 2016 survey introduced variables related to internet access, key mediating variables were missing from that year’s dataset. The 2018 wave included 11,418 respondents and the 2020 wave included 11,398 respondents. Our final analytical sample was derived through the following steps: (1) we retained households that participated in both survey waves to create a balanced panel; (2) we excluded observations with missing data on internet access, the CES-D scale, and core demographic covariates. The final analytical sample consisted of *N* = 22,800 respondents.

### 3.2 Variables and measures

#### 3.2.1 Dependent variables

The dependent variable in this study is the mental health of the older adults. Current academic measures of mental health in the older adults mainly include depression, loneliness, and life satisfaction (41, 42), among which the degree of depression is widely used in measuring mental health (43, 44). In this study, depressive symptoms serve as the primary indicator of mental health. This choice is the standard outcome measure commonly adopted in research on digital technology and aging, ensuring comparability with prior empirical evidence (45, 46). Depression levels were measured using the CES-D scale within the CLASS questionnaire. The CES-D scale was selected for its well-established reliability and validity in gerontological research. Its Chinese version has been validated extensively and is recognized as an appropriate instrument for assessing depressive symptoms among older Chinese older adults (25, 47). This scale comprises nine questions concerning the respondents’ feelings and behaviors over the past week, including six negative and three positive items. Each item has three response options: 1 (not at all), 2 (sometimes), and 3 (frequently). The total depression score is calculated by summing the scores from all nine items, with items 1, 4, and 9 reverse-scored. The total score ranges from 9 to 27, with higher scores indicating poorer mental health among the older adults.

To comprehensively assess the impact of internet access on the mental health of the older adults, this study will also examine its effects on feelings of loneliness and life satisfaction in robustness tests. This approach is supported by extensive literature demonstrating strong associations between these variables and overall psychological wellbeing (41, 48, 49). The specific procedures are as follows: Loneliness is transformed into a dummy variable, where 0 represents not lonely and 1 represents lonely. Life satisfaction is measured based on the questionnaire item “Overall, how satisfied are you with your current life?” Responses are coded as 5 for very satisfied, 4 for relatively satisfied, 3 for neutral, 2 for relatively dissatisfied, and 1 for very dissatisfied. After reverse-coding this item, higher final values signify greater life satisfaction.

#### 3.2.2 Independent variables

The independent variable in this study is internet access. At the micro-individual level, Internet usage serves as a key measure of Internet access (50, 51). Following the existing literature, this variable is constructed based on the CLASS questionnaire item, “Do you use the internet (including accessing it via mobile devices)?” Responses indicating “never use the internet” are assigned a value of 0, while responses of “a few times a year,” “at least once a month,” “at least once a week,” and “every day” are all assigned a value of 1, coded as internet users. Furthermore, this study will also explore the effects of internet social functionality usage among the older adults on mental health in robustness tests. Social interaction represents a primary and psychologically salient dimension of internet access among the older adults (52). Replacing the overall measure of internet access with this more focused indicator helps reduce potential measurement error and verify the stability of the main results.

#### 3.2.3 Control variables

This study includes multiple control variables based on Grossman’s health demand theory and relevant empirical aging research to address potential confounding factors that may influence the older adults’s mental health. These variables include age, marital status (0 = widowed, divorced, or unmarried; 1 = married), employment status (0 = unemployed, 1 = employed), the number of chronic diseases, the number of Activities of Daily Living (ADL) impairments, the number of Instrumental Activities of Daily Living (IADL) impairments, income, the number of children, the average age of children, and whether live with children (0 = no, 1 = yes). Table 1 shows the description and statistics of these variables.

**Table 1:** Descriptive statistics of the variables.

Variable	Definition	Observations	Mean	Std. Dev.	Max	Min
Dependent variables						
CES-D	CES-D scale (9–27 points), the higher the score, the higher risk of depression	22,800	15.719	3.025	9	27
Life satisfaction	Five-point scale (1–5), the higher the score the higher the level of satisfaction	22,605	3.75	0.882	1	5
Loneliness	Loneliness = 1, otherwise = 0	22,805	0.447	0.497	0	1
Independent variables						
ICT	If use internet = 1, otherwise = 0	22,811	0.228	0.42	0	1
Social media use	If using social media = 1, otherwise = 0	5,203	0.908	0.289	0	1
Control variables						
Age	Years of age	22,811	71.52	6.997	60	108
Marriage	If married = 1, otherwise = 0	22,811	0.723	0.447	0	1
Work	If work = 1, otherwise = 0	22,811	0.253	0.435	0	1
Chronic	Number of chronic diseases	22,811	1.484	1.484	0	22
ADL	Number of activities of daily living (ADL) impairments	22,811	0.315	1.042	0	7
IADL	Number of activities of daily living (IADL) impairments	22,811	0.744	1.939	0	10
Income	Log of personal income	22,762	8.451	1.258	4.096	12.919
Family size	Numbers of children	22,811	2.468	1.363	0	10
Child age	Average age of children	22,107	44.717	7.64	1	77
Living with children	Whether living with children, yes = 1, otherwise = 0	22,811	0.337	0.473	0	1
Friend network	Number of friends members available for support (scored 0–15)	22,811	6.515	3.135	0	15
Family network	Number of family members available for support (scored 0–15)	22,811	7.389	2.748	0	15
Social participation	Participated = 1, otherwise = 0	22,811	0.322	0.467	0	1
Political participation	Participated = 1, otherwise = 0	22,811	0.426	0.495	0	1
Older adults facility	If older adults care facilities exist = 1, otherwise = 0	22,811	0.69	0.463	0	1
Care	Frequency of receiving housework help from children	22,119	2.282	1.409	0	6
Meet	Frequency of in-person meetings with children	22,119	2.933	1.071	0	4

CES-D refers to the Center for Epidemiological Studies Depression Scale (62), a commonly used instrument for assessing depressive symptoms.

#### 3.2.4 Channel and constraint variables

The channel variables selected for this study are social networks. Drawing on Lubben’s (53) research, personal networks are operationalized through two dimensions: family networks and friend networks. These networks are measured using six items from the CLASS questionnaire. Specifically, the questionnaire assesses the number of friends and family members the older adults could directly contact, confide in, and rely on for help. Response options included none, 1 person, 2 people, 3 to 5 people, 5 to 8 people, and 9 or more, assigned values of 0, 1, 2, 3, 4, and 5, respectively. The scores for the three questions related to each network type were summed, resulting in a family network score and a friend network score, each ranging from 0 to 15. Higher scores indicate larger network sizes. Community networks are measured by focusing on social participation. Social participation is divided into two categories: voluntary participation and political participation. Voluntary participation is measured through the survey question “In the past year, how frequently have you participated in the following activities?” which lists eight types of volunteer activities; participation in any one activity indicates voluntary engagement. Political participation is assessed based on responses to the survey question “Have you participated in local neighborhood committee/village committee voting elections in the past three years?” with a “yes” response indicating political participation.

Furthermore, this study incorporates community-based older adults care organizations and facilities, as well as offline companionship and care from children, as constraint variables to examine how the offline care environment influences the outcomes of the older adults’s internet access.

### 3.3 Empirical model

To assess the impact of internet access on the mental health of the older adults, the following econometric model (Equation 1) was constructed:


$${\text{MentalHealth}}_{\mathrm{it}}=\alpha +\beta {\mathrm{ICT}}_{\mathrm{it}}+\gamma X_{\mathrm{it}}+\mu_{i}+\tau_{t}+\varepsilon_{\mathrm{it}}$$ (1)

Where i and t denote the individual and year, respectively. The dependent variable, ${\text{MentalHealth}}_{\mathrm{it}}$ represents the mental health level of older adult i at time t . The key independent variable, ${\mathrm{ICT}}_{\mathrm{it}}$ denotes whether older adults individual i has internet access during period t . $X_{\mathrm{it}}$ denotes the control variables. The term $\mu_{i}$ represents individual fixed effects. The term $\tau_{t}$ denotes time fixed effects, while $\varepsilon_{\mathrm{it}}$ specifies the random error term.

This paper employs a fixed-effects model primarily to mitigate endogeneity problems arising from omitted unobservable variables. At least two types of key variables may be omitted: first, personality traits, such as how more outgoing individuals may be more likely to use the internet and also have better psychological states, which are challenging to capture in traditional cross-sectional data; second, family-level factors, such as the quality of family interactions, which may influence both the frequency of internet access and the psychological state of the older adults. Omitting these variables may lead to biased estimates. Although individual personality and family environment are difficult to measure, these characteristics are largely time-invariant for individuals over the short term. By controlling for unobserved, time-invariant characteristics at the individual level, the fixed-effects model effectively mitigates the endogeneity problem caused by such omitted variables, thereby improving the reliability of the estimation results.

## 4 Results

### 4.1 Descriptive analysis

Table 2 presents the results of independent samples *t*-tests, which were used as preliminary analyses to examine unadjusted between-group differences in the older adults who have internet access and those who do not. The results indicate that Internet users were significantly better than non-users in terms of mental health and social connectedness. Specifically, Internet users reported significantly lower levels of depression, less loneliness, and greater life satisfaction. In addition, they participated in social activities significantly more often and had larger social networks. These descriptive findings provide initial support for the hypothesis that Internet access is associated with improved mental health and enhanced social resources among the older adults, and provide a foundation for further research based on regression analysis.

**Table 2:** Mean differences between internet users and non-users.

Variable	Non-users (ICT = 0) (1)	Users (ICT = 1) (2)	Difference (2)–(1)
CES-D	16.077	14.509	−1.568***
Life satisfaction	3.704	3.903	0.199***
Loneliness	0.474	0.355	−0.119***
Social participation	0.299	0.398	0.099***
Political participation	0.418	0.452	0.034***
Family network	7.324	7.609	0.285***
Friend network	6.352	7.064	0.712***

Columns (1) and (2) present the mean values for each group. Column (3) presents the mean difference (Column 2 – Column 1). A *t*-test was performed on this difference, and the significance levels are reported. ***p < 0.01, **p < 0.05, *p < 0.1.

### 4.2 Baseline regression results

#### 4.2.1 Basic results

Table 3 presents the baseline regression results for the impact of internet access on the mental health of the older adults. The results in column (1) indicate that the coefficient for internet access is negative and statistically significant at the 1% level, suggesting that without controlling for other variables, the older adults who use the internet have significantly lower depression scores. In column (2), after adding control variables, the model’s explanatory power improved, with the R-squared increasing from 0.011 to 0.046, while the effect of internet access remained significantly negative. Since higher CES-D scores indicate poorer mental health, the significantly negative coefficients demonstrate that internet access is associated with better mental health among the older adults. Specifically, internet users exhibit depression scores approximately 0.91 points lower than non-users.

**Table 3:** Effects of internet access on mental health of the older adults.

Variable	(1)	(2)
	CES-D	CES-D
ICT	−0.868*** (0.129)	−0.915*** (0.130)
Age		0.074 (0.055)
Marriage		−0.334*** (0.126)
Work		−0.311** (0.126)
Chronic		0.157*** (0.038)
ADL		0.283*** (0.075)
IADL		0.043 (0.041)
Income		−0.036* (0.021)
Family size		−0.443** (0.190)
Child age		0.017* (0.010)
Live with children		−0.093 (0.112)
Individual FE	YES	YES
Year FE	YES	YES
Observations	22,800	22,055
R-squared	0.011	0.046

Robust standard errors in parentheses; ***p < 0.01, **p < 0.05, *p < 0.1.

The results for the other control variables were generally consistent with theoretical expectations and empirical evidence. The older adults with spouses exhibited significantly lower levels of depression compared to those without spouses, which aligns with anticipated outcomes. Regarding health status, the number of chronic diseases and limitations in activities of daily living (ADL) were associated with significantly higher depression levels among the older adults. This suggests that the decline in physiological function directly exacerbates the deterioration of mental health, a finding consistent with existing literature. In terms of economic and labor participation, working older adults individuals showed better mental health than their non-working counterparts, and higher income was also associated with better mental health. This discrepancy may arise because employment fosters social engagement and self-identity, while higher income directly elevates living standards, collectively contributing to improved psychological wellbeing.

### 4.3 Robustness checks

This section examines the robustness of the baseline regression results through adjustments to the dependent variable, the key independent variable, and by employing propensity score matching (PSM) and an instrumental variable approach.

#### 4.3.1 Adjustment for dependent variable

To comprehensively assess the mental health of the older adults, we substituted life satisfaction and loneliness for the original dependent variable, depression. Life satisfaction, being an ordered categorical variable, was estimated using both fixed-effects models and panel ordered probit models. Loneliness, a binary variable, was estimated using fixed-effects models and panel probit models. The estimation results are presented in Table 4. The findings, which were consistent across different dependent variables and estimation methods, indicate that internet access significantly enhances life satisfaction and reduces loneliness among the older adults. These results align with the baseline regression, hence reinforcing the robustness of the findings.

**Table 4:** Robustness checks for the effects of Internet access on mental health.

Variable	(1)	(2)	(3)	(4)
	Alternative dependent variable	Alternative dependent variable	Alternative independent variable	PSM K-nearest (ATT)
	Life satisfaction	Loneliness	CES-D	CES-D
ICT	0.211*** (0.037)	−0.089*** (0.021)		−1.376*** (0.064)
Social media use			−0.982*** (0.134)	
Controls	YES	YES	YES	YES
FEs	YES	YES	YES	YES
Observations	21,874	22,058	22,055	21,874
*R*-squared	0.090	0.019	0.047	—

(1) Standard errors are reported in parentheses. (2) Columns (1)–(2) use alternative dependent variables (Life satisfaction and Loneliness); Column (3) uses an alternative independent variable (Social media use); Column (4) presents the Average Treatment Effect on the Treated (ATT) using K-nearest neighbor matching. (3) ***p < 0.01, **p < 0.05, *p < 0.1.

#### 4.3.2 Adjustment for independent variable

To further validate the impact of internet access on the mental health of the older adults, we conducted an additional robustness test by substituting the core independent variable. Specifically, we replaced the measure of overall internet use with a variable capturing the use of internet-based social features (e.g., voice and video calls, text-based chat). General internet access encompasses a range of activities, including information retrieval, news browsing, and social engagement. The use of social functions typically involves bidirectional communication, which entails an exchange of psychological resources and may therefore exert a more direct influence on mental health. In contrast, activities such as news browsing are predominantly unidirectional and likely have weaker effects on mental wellbeing. By focusing on social feature use, we can more precisely identify the specific online behaviors that influence mental health, thereby enhancing the explanatory power of the results. The results in Table 4 indicate that the older adults who use internet social features exhibit lower levels of depressive symptoms. This finding aligns in direction and significance with the baseline regression coefficient, thus confirming the robustness of the initial results.

#### 4.3.3 PSM

This study utilizes Propensity Score Matching (PSM) to mitigate self-selection bias in internet access among the older adults. Robustness tests were conducted using three methods: k-nearest neighbor matching within calipers, caliper matching, and local linear regression matching. Table 4 presents the Average Treatment Effect on the Treated (ATT) of internet access on the mental health of the older adults, as estimated by the three matching methods. The results indicate that the ATT values are −1.376, −1.488, and −1.388, respectively, with the absolute values of the T-statistics being significant at the 1% level. Although the ATT values vary slightly across the three methods, their signs are highly consistent, further validating the robustness of the baseline regression results.

#### 4.3.4 Instrumental variable estimation

Despite the fixed-effects approach, the baseline regression estimates might still suffer from bias due to reverse causality or omitted variables. On the one hand, the older adults with better mental health, potentially due to stronger cognitive abilities or a more optimistic outlook, may be more likely to have internet access, creating reverse causality. On the other hand, although the model controls for observable variables such as family and child characteristics and physical health status, other unobserved time-varying characteristics may simultaneously affect internet access and mental health. Therefore, this study employs an instrumental variable (IV) approach and two-stage least squares (2SLS) estimation to address endogeneity and test the robustness of the baseline results.

This study selects “communication expenditure” and “community-level proportion of internet access” as instrumental variables. Communication expenditure exhibits a strong correlation with internet access behavior and can reflect the cost basis for internet access and utilization; however, it does not directly impact older adults mental health, thereby satisfying the exogeneity condition. The proportion of internet access by others within the community can reflect the degree of internet diffusion. Generally speaking, the higher the proportion of internet access by others in the community, the greater the likelihood that older adults individuals will use the internet. Meanwhile, internet diffusion does not influence older adults mental health, similarly satisfying the exogeneity condition for instrumental variables. Table 5 reports the IV estimation results. The instrumental variables pass the weak instrument test (first-stage F-statistic = 2916.93) and the over-identification test, indicating they are both strong and exogenous. The second-stage regression results show that the effect of internet access on depression remains negative and significant, aligning with the baseline findings. The absolute value of the coefficient is larger than in the baseline regression, suggesting that the initial estimates may have underestimated the actual effect of internet access on the mental health of the older adults. This finding further validates the robustness of the baseline results.

**Table 5:** Results of instrumental variable (IV) estimation.

Variable	(1)	(2)
	ICT	CES-D
ICT		−2.718*** (0.115)
IV		
communication expenditure	0.032*** (0.003)	
Community-level proportion of internet access	0.820*** (0.012)	
Control variables	YES	YES
IV test		
Observations	16,772	16,772
*R*-squared	0.393	0.057
*F*-statistic	2,916.93***	
Cragg–Donald Wald *F* statistic	3,081.73	
Stock–Yogo weak ID test critical value (10%)	19.93	
Endogeneity test (Durbin–Wu–Hausman *χ*^2^, *p*-value)		208.13 (0.000)
Overidentification test (Sargan *χ*^2^, *p*-value)		1.47 (0.225)

Robust standard errors in parentheses; ***p < 0.01, **p < 0.05, *p < 0.1.

### 4.4 Mechanisms analysis

#### 4.4.1 Mediating effects of social networks

Based on the theoretical analysis, this study examines the effects of internet access on the family networks, friend networks, and social participation of the older adults. In the econometric models, the dependent variables are the size of family and friend networks, as well as social participation status. The results presented in Table 6. The results indicate that the internet access has a significant positive effect on the friend networks of the older adults (column (1) of Table 6), while no such effect on their family networks. This disparity suggests that the internet access of the older adults significantly expands their friend networks, whereas its effect on family networks is comparatively limited. We can attribute this differential impact to the greater potential for expansion within friend networks. The older adults can leverage the internet to proactively connect with former acquaintances or forge new friends, particularly as they experience reduced offline social opportunities post-retirement. Conversely, family networks are subject to rigid constraints, such as the number of children, which limit their potential for expansion. Internet access primarily serves to maintain or increase the frequency of contact with children, rather than generating significant network growth. Furthermore, within the context of traditional filial piety, filial visits are often perceived as obligatory, whereas friend networks rely more on active communication. Internet access effectively reduces the costs associated with such active engagement. Consequently, internet access acts as a catalyst for the expansion of friend networks while primarily serving a stabilizing function within family networks. This conclusion implies that internet access can augment the size of the older adults’s social networks, thereby contributing to the improvement of their mental health.

**Table 6:** Effects of Internet access on social networks of the older adults.

Variables	Friend network	Family network
ICT	0.323*** (0.121)	0.165 (0.110)
Control variables	YES	YES
Individual FE	YES	YES
Year FE	YES	YES
Observations	22,062	22,062
*R*-squared	0.029	0.039

Robust standard errors in parentheses; ***p < 0.01, **p < 0.05, *p < 0.1.

The results presented in Table 7 indicate that internet access has a significant positive effect on both voluntary and political participation among the older adults. This effect may be attributed to the internet’s capacity to overcome barriers to social engagement in this demographic directly. On one hand, online platforms facilitate real-time interactions, thereby expanding offline social networks and promoting volunteer activities. On the other hand, the internet enables older adults to conveniently access political information, which in turn enhances their willingness to engage in public affairs. This dual effect substantially increases the likelihood of social participation among the older adults, a factor that has been shown in multiple studies to alleviate feelings of loneliness (54, 55). Consequently, internet access improves the mental health of the older adults by strengthening their social engagement.

**Table 7:** Effects of internet access on social participation of the older adults.

Variable	(1)	(2)
	Voluntary participation	Political participation
ICT	0.032* (0.019)	0.091*** (0.021)
Control variables	YES	YES
Individual FE	YES	YES
Year FE	YES	YES
Observations	22,062	22,062
*R*-squared	0.030	0.023

Robust standard errors in parentheses; ***p < 0.01, **p < 0.05, *p < 0.1.

#### 4.4.2 Analysis of constraining factors

Based on the theoretical framework, community activity facilities and the frequency of family care are selected as moderating variables to examine how family and community older adults care environments influence the effect of internet access. Interaction terms between internet access and these moderating variables were constructed to test how offline care environments shape the outcomes of internet use among the older adults.

As presented in Table 8, the results of Column (1) indicate that the interaction term between internet access and the presence of older adults care service facilities has a significantly negative coefficient. This finding suggests that the depression-alleviating effect of internet access is stronger for the older adults residing in communities with such facilities.

**Table 8:** Results of constraints factor estimations.

Variable	(1) CES-D	(2) CES-D	(3) CES-D
ICT	−0.150 (0.264)	−1.403*** (0.392)	−1.301*** (0.253)
Older adults facility	−0.361*** (0.113)		
Meet		−0.286*** (0.053)	
Care			−0.179*** (0.040)
ICT × Facility	−1.014*** (0.293)		
ICT × Meet		0.168 (0.120)	
ICT × Care			0.160* (0.084)
Control variables	YES	YES	YES
Individual FE	YES	YES	YES
Year FE	YES	YES	YES
Observations	22,055	22,055	22,055
*R*-squared	0.054	0.052	0.050

Robust standard errors in parentheses; ***p < 0.01, **p < 0.05, *p < 0.1.

Column (2) and (3) of Table 8 present the results for the interaction terms between internet access and the frequency of children’s visits and the level of care provided by children, respectively. The results indicate that the higher the frequency of meeting with children or the frequency of care provided by children, the lower the depression scores among older adults. The coefficients of both interaction terms indicate that the more frequent visits and a higher level of care from children diminish the effectiveness of internet access in reducing depression. This pattern can be attributed to the compensatory role of children’s care, which may partially substitute for the psychological benefits derived from internet access. Therefore, the effect of internet access on alleviating depression is dynamically moderated by family resources and community environments.

### 4.5 Heterogeneity analysis

Our prior analysis demonstrated that internet access alleviates depressive symptoms among the older adults, thereby enhancing their mental wellbeing. However, the impact varies across different subgroups of the older adults population. Therefore, we conducted a more in-depth examination of the impact of internet access on the mental health of older adults individuals, taking into account both residential location and age. Initially, we categorized the older adults sample into urban and rural groups based on their residential locations. We employed a group-wise regression approach to conduct a heterogeneity test. We utilized the Fisher test to determine if there were significant differences in the regression coefficients between the two groups. The results, presented in Table 9, indicate that internet access positively and significantly impacts the mental health of the older adults in both urban and rural settings. However, a comparison of the regression coefficient estimates reveals that the promotion of mental health through internet access is more pronounced among urban older adults individuals. The *p*-value for the coefficient difference is 0.040, indicating a statistically significant difference between the two groups. This heterogeneity may be attributed to the higher digital literacy and richer online resources available to urban older adults individuals. Conversely, rural areas may face limitations due to regional infrastructure or technical barriers, thus hindering the full realization of the potential benefits of internet access.

**Table 9:** Results of heterogeneity analysis estimation.

Variable	(1)	(2)	(3)	(4)
	Urban	Rural	60–80	≥80
	CES-D	CES-D	CES-D	CES-D
ICT	−0.986*** (0.176)	−0.547** (0.235)	−0.912*** (0.135)	−0.451 (0.672)
Individual FE	YES	YES	YES	YES
Year FE	YES	YES	YES	YES
Control variables	YES	YES	YES	YES
*R*-squared	0.048	0.038	0.045	0.068
Observations	11,615	10,440	18,721	3,334
*p*-value	0.040**		0.020**	

Robust standard errors in parentheses; ***p < 0.01, **p < 0.05, *p < 0.1.

Furthermore, following prior gerontological studies that commonly adopt 80 years as a threshold for the oldest-old category (56, 57), we divided the older adults sample into two age groups: young-old (60–80 years) and old-old (80 years and older). This cutoff is also supported by empirical evidence that the prevalence of functional limitations, multiple chronic conditions, and cognitive impairment rises substantially after age 80, marking a distinct phase in the aging process. We employed a similar group-wise regression approach to examine the differences and utilized the Fisher test to assess the significance of the differences between the two groups. Table 9 reveals that the positive effect of internet access on mental health is more substantial for the younger older adults group. The p-value for the coefficient difference is 0.020, indicating a statistically significant difference between the two groups. As individuals age, they may experience multiple impairments, such as cognitive decline and visual impairments, which can reduce the frequency and quality of internet access. Consequently, internet access has a limited impact on enhancing the mental health of individuals aged 80 and above.

## 5 Discussion

Drawing on nationally representative data from the 2018–2020 China Longitudinal Aging Social Survey (CLASS), this study examines how internet access shapes the mental health of the older adults and, critically, through which relational channels and under what contextual conditions these benefits materialize. The analysis enriches and extends existing research in the following three key areas.

Our principal finding that internet access significantly reduces depressive symptoms among the older adults. This finding is robust to a series of rigorous tests, including alternative measures of explanatory and dependent variables, propensity score matching (PSM), and an instrumental variable approach. This result not only corroborates existing evidence that digital technology alleviates depressive symptoms through enhanced psychological connectivity (10, 58) but also contextualizes it within China’s rapid population aging.

Mechanism analysis reveals that social networks and social participation mediate the relationship between internet access and mental health, a pattern consistent with prior research emphasizing the role of digital connectivity in sustaining emotional bonds (59). By contrast, our study distinguishes between friend networks, which are voluntary, actively maintained through communication, and characterized by relational reciprocity, and family networks, which are obligatory, constrained by kinship structure, and governed by normative expectations. We demonstrate that the mental health benefits of internet access are transmitted through differentiated network channels, with distinct implications for psychological wellbeing. Notably, internet access has a more substantial effect on expanding friend networks than family networks. This is because family ties are often constrained by kinship structures and are less malleable, whereas the internet plays a pronounced role in broadening more voluntary friend ties (40). This finding implies internet access does not merely “reduce loneliness” or “increase social support” in a generic sense; rather, it reconfigures the composition and reach of older adults’ relational ties through differentiated pathways. This finding clarifies how digital participation can enhance psychological wellbeing through specific relationship channels.

Furthermore, our study is the first to reveal that the mental health gains from internet access are significantly conditioned by the interplay of family care and community support resources. The accessibility of community activity facilities amplifies the positive impact of internet access, as comprehensive community environments create complementary effects with online activities—for instance, by extending virtual interactions into offline engagement. Conversely, frequent family caregiving attenuates the mental health benefits of internet access, aligning with substitution effect theory; ample offline support may reduce the perceived need for online alternatives. This finding demonstrates that the benefits of internet access are not uniform but depend on the offline caregiving and community contexts in which older adults are embedded. Therefore, maximizing the health benefits of internet access depends on integrating online and offline support systems. Communities should provide settings that facilitate digital technology use, while families should encourage autonomous internet access rather than providing substitutive support.

Subgroup heterogeneity confirms well-documented patterns in the digital aging literature. Urban residents derive larger mental health benefits from internet access than rural residents, mirroring documented urban–rural disparities in digital infrastructure and literacy (25, 60). Younger older adults individuals (aged 60–80) benefit significantly, but the oldest-old (over 80) show no marked improvement, aligning with the technology adoption gradient in cross-national aging studies (61). These patterns underscore a boundary condition: internet-based mental health interventions are not universally scalable. For the oldest-old, compounded barriers of physical limitation, cognitive decline, and digital unfamiliarity may block the digital pathway to psychological wellbeing. Policies should remain multimodal, incorporating non-digital interventions such as community-based social prescribing and home visitation programs for populations where digital connectivity yields diminishing returns.

Several limitations should be acknowledged. First, the data cover the period from 2018 to 2020, which includes the onset of the COVID-19 pandemic, a period that markedly accelerated digital technology adoption among the older adults. The observed patterns may therefore not fully represent digital engagement among the older adults in the post-pandemic era, and future research could examine whether the network-based mechanisms identified in this study persist or evolve as the digital landscape continues to change. Second, depressive symptoms were self-reported and may be subject to reporting bias; future studies incorporating clinical diagnostic measures would strengthen the evidence base. Third, while the CLASS survey provides a nationally representative sample of China, the findings may have limited generalizability beyond the Chinese context. Future cross-national comparative research could further illuminate how contextual factors shape the relationship between internet access and mental health.

## 6 Conclusion

Based on nationally representative survey data, this study finds that internet access serves as a significant positive factor enhancing the psychological wellbeing of the older adults. The primary mechanisms are the strengthening of social networks, particularly with friends, and the promotion of social participation. However, the magnitude of this effect is significantly moderated by community resources and family care, and exhibits marked heterogeneity across residential locations and age cohorts. These results validate and extend the technological protection hypothesis and digital divide theory, enriching the theoretical framework of healthy aging from the perspective of China’s rapidly aging population. Future policy should focus on age-friendly digital adaptation, coordinated family-community support, and targeted interventions for disadvantaged subgroups to fully unlock the welfare potential of digital technologies, offering valuable insights for aging societies worldwide.

## Data Availability

The data analyzed in this study is subject to the following licenses/restrictions: Restricted access: data are available only upon formal application and permission from the China Longitudinal Aging Social Survey (CLASS) data management team. No unrestricted public access. Requests to access these datasets should be directed to zhangchi@zufe.edu.cn.

## Footnotes

1 International statistical agencies commonly use age 65 as the threshold for defining the older adults. China’s national aging surveys, including CLASS, adopt age 60 as the sampling criterion, xx consistent with the legal definition stipulated in China’s Law on the Protection of the Rights and Interests of the older adults.
